# Genetic Aberrations of DNA Repair Pathways in Prostate Cancer: Translation to the Clinic

**DOI:** 10.3390/ijms22189783

**Published:** 2021-09-10

**Authors:** Aruni Ghose, Michele Moschetta, George Pappas-Gogos, Matin Sheriff, Stergios Boussios

**Affiliations:** 1 Barts Cancer Centre, Department of Medical Oncology, St. Bartholomew’s Hospital, Barts Health NHS Trust, W Smithfield, London EC1A 7BE, UK; aruni.ghose@nhs.net; 2 Faculty of Life Sciences & Medicine, King’s College London, London WC2R 2LS, UK; 3 CHUV, Lausanne University Hospital, Rue du Bugnon 21, CH-1011 Lausanne, Switzerland; michelemoschetta1@gmail.com; 4 Department of Surgery, University Hospital of Ioannina, 45111 Ioannina, Greece; pappasg8@gmail.com; 5 Department of Urology, Medway NHS Foundation Trust, Windmill Road, Gillingham, Kent ME7 5NY, UK; matin.sheriff@nhs.net; 6 Department of Medical Oncology, Medway NHS Foundation Trust, Windmill Road, Gillingham, Kent ME7 5NY, UK; 7 Faculty of Life Sciences & Medicine, School of Cancer & Pharmaceutical Sciences, King’s College London, London SE1 9RT, UK; 8 AELIA Organization, 9th Km Thessaloniki–Thermi, 57001 Thessaloniki, Greece

**Keywords:** prostate cancer, DNA damage repair, PARP, BRCA, next-generation sequencing

## Abstract

Prostate cancer (PC) is the second most common cancer in men worldwide. Due to the large-scale sequencing efforts, there is currently a better understanding of the genomic landscape of PC. The identification of defects in DNA repair genes has led to clinical studies that provide a strong rationale for developing poly (ADP-ribose) polymerase (PARP) inhibitors and DNA-damaging agents in this molecularly defined subset of patients. The identification of molecularly defined subgroups of patients has also other clinical implications; for example, we now know that carriers of breast cancer 2 (*BRCA2*) pathogenic sequence variants (PSVs) have increased levels of serum prostate specific antigen (PSA) at diagnosis, increased proportion of high Gleason tumors, elevated rates of nodal and distant metastases, and high recurrence rate; *BRCA2* PSVs confer lower overall survival (OS). Distinct tumor PSV, methylation, and expression patterns have been identified in *BRCA2* compared with non-*BRCA2* mutant prostate tumors. Several DNA damage response and repair (DDR)-targeting agents are currently being evaluated either as single agents or in combination in patients with PC. In this review article, we highlight the biology and clinical implications of deleterious inherited or acquired DNA repair pathway aberrations in PC and offer an overview of new agents being developed for the treatment of PC.

## 1. Introduction

Prostate cancer (PC) is the second most common neoplasm among men [1,2]. According to Cancer Research United Kingdom (UK) (https://www.cancerresearchuk.org/health-professional/cancer-statistics/statistics-by-cancer-type/prostate-cancer#heading-Zero, accessed on 26 May 2021) it is the second leading cause of cancer- related death in the UK [3]. Locally advanced disease is curable, although metastatic disease has limited therapeutic options. Androgen Receptor (AR) signaling represents still the most important pathway to target for developing new and more effective therapies, and androgen deprivation therapy (ADT) is still the cornerstone of management of PC patients. Resistance development to ADT defines the status of metastatic castration resistant prostate cancer (mCRPC) still associated with dismal clinical outcome, poor prognosis and limited therapeutic options [2,4,5].

PC is known for its genomic instability [6]. Almost 15% of mCRPC cases lack AR expression, predominantly neuroendocrine PC and double-negative PC, which belong to the Androgen Receptor null and Neuroendocrine Receptor null phenotype [2,6]. It has been shown that 90% of mCRPCs harbor clinically actionable germline and somatic alterations in non-AR related pathways [2,4,5,6], mostly DNA damage response (DDR) defects, which represent 25% of these alterations [2]. DDR genes are involved in preserving cell genomic stability, repairing DNA aberrations during cell cycle, ensuring a correct mitotic cell division, and distribution of the genomic material to the daughter cells [2,7].

DDR gene dysfunction, either inherited or acquired, leads to genomic instability and higher mutation rate and hence enhanced tumorigenesis and intra-tumor heterogenecity [2,7].

DDR pathways can be seen as a network of cellular mechanisms, which employ surveillance proteins to sense DNA integrity, signal damage by activating cell cycle checkpoints and promote its repair [7]. DNA damage may be endogenous [(spontaneous hydrolytic and oxidative reactions of DNA with water and reactive oxygen species] or exogenous (reactions facilitated by environmental, physical and chemical agents like ultraviolet (UV) radiation or alkylating agents). This damage may be in the form of single- stranded break (SSB) or double-stranded break (DSB) [8,9].

**Figure 1:**
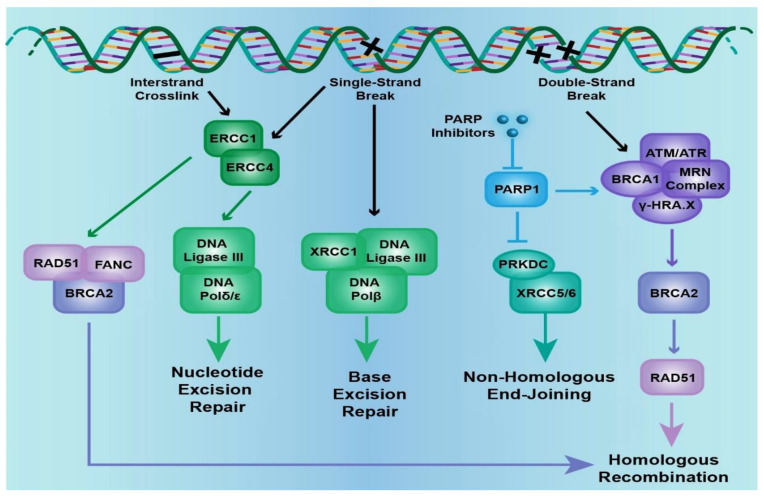
The cell signaling pathways that are involved in DNA damage. Excision repair cross-complementation group genes (*ERCC-1/-4*), X-ray repair complementing defective repair in Chinese hamsters’ cell genes (*XRCC-1/-5/-6*), poly(ADP-ribose) polymerase 1 (*PARP1*), the Fanconi anemia gene complex (*FANC-A/-C/-D2/-E/-F/-G/-L*), Rad51 recombinase (*RAD51*), ATM serine/threonine kinase (*ATM*), ATR serine/threonine kinase (*ATR*), protein kinase, DNA-activated catalytic polypeptide (*PRKDC*), and the breast cancer, early onset genes (*BRCA1/2*) are included in the DNA damage pathway. Specific nodes in the pathway that are therapeutically actionable are mentioned (adapted from https://www.mycancergenome.org/).

As mentioned above, the cell has several ways to repair DNA damage; among those, base excision repair (BER) involves removal of small, non-helix-distorting base lesions resulting from deamination, oxidation, alkylation reactions, while nucleotide excision repair (NER) removes bulky, helix-distorting nucleotide lesions resulting from UV radiation and chemotherapeutic agents. Mismatch repair (MMR) removes insertion, deletion and misincorporation of bases resulting from DNA replication and recombination errors that have escaped proofreading [10]. DSB repair involves homologous recombination (HR) and non-homologous end joining (NHEJ) pathways (Figure 1) [11]. HR involves template dependent repair; undamaged homologous chromatid or chromosome is used as template for repair and is therefore operable during S/G2 phase of cell cycle. It leads to reconstitution of original sequence and therefore usually error-free. NHEJ modifies and ligates broken ends without a template and can function all throughout the cell cycle: template independent repair. It can be error prone as it can generate deletions or insertions, without regard for homology [12].

*BRCA* genes are the most frequently mutated DDR gene in PC and are a significant part of the HR pathway. Table 1 depicts single-nucleotide polymorphisms and germline DDR gene mutations in PC.

### 1.1. DNA Repair Mutations in Prostate Cancer

The incidence of germline mutations in DDR genes among men with mCRPC varies between 11–33% making it significantly higher than that of localized disease [13]. As previously mentioned, the commonest DDR aberration is *BRCA2*, followed by *CDK12*, *ATM*, *CHEK2*, *BRCA1*, *MSH2*, *FANCA*, *MLH1*, and *RAD51* [2]. The most frequent somatic genomic aberrations include *AR* (62.7%), *ETS* family (56.7%), *TP53* (53.3%), and *PTEN* (40.7%) [4].

The breast cancer genes 1 and 2 (*BRCA1* and *BRCA2*) are located at chromosome 17q21 and 13q12, respectively [14]. They are large genes consisting of 100 and 70 kb, respectively [15]. They have an autosomal dominant inheritance pattern with incomplete penetrance [16]. They are part of an HR DNA repair pathway usually utilized for DSB repair. *BRCA* dysfunction determines HR deficiency, which is usually compensated by NHEJ, an error prone repair system [15]. In any case of impairment of HR, synthetic lethality induced by poly (ADP-ribose) polymerase (PARP) inhibition occurs and may target tumor tissue selectively. The synthetic lethality could even represent the therapeutic strategy of cancers with *BRCA*-like properties, known as “BRCAness” [8]. This is based on the observation that deficiency in genes beyond *BRCA* that are also implicated in HR may confer sensitivity to PARP inhibitors. Consequently, alterations in DDR genes, particularly in those involved in HR repair, are predictors of response to PARP inhibition [5].

Structurally speaking, although both *BRCA* genes have a nuclear localization sequence, their functional domains hardly display homology. The *BRCA2* gene has eight internal repeats also known as BRC repeats and a DNA binding domain which interact with *RAD51* and *DSS1* (deleted in split-hand/split foot protein 1) respectively, both of which are HR-related proteins. *BRCA1* has three domains: RING, coiled coil, and BRCT which interact with BARD1 (*BRCA1*-associated RING domain), PALB2 (partner and localizer of *BRCA2*), *ABRA1* (abraxas), *CtIP* (CtBP interactive protein), and *BRIP1* (BRCA1-interacting protein *C*-terminal helicase 1). Hence *BRCA1* is a major component of the HR, but apart from that, is also involved in DNA damage sensing, cell cycle regulation, E3 ubiquitin ligase activity and chromatin remodeling [15].

Incidence of germline *BRCA* mutations in newly diagnosed PC is 1.2–2% [17]. *BRCA1/2* carriers can have around 4- and 8-fold risk of developing PC, respectively [16]. Moreover, *BRCA* mutation carriers with localized PC have worse outcomes than those who are wild type, regardless of the local treatment they have previously undergone. Indeed, *BRCA* carriers have the worst prognosis, higher Gleason Score (8+), increased rate of lymph node involvement, earlier onset of distant metastasis, and shorter survival [17]. Patel et al. identified no statistically significant associations between *BRCA1* pathogenic sequence variants (PSVs) and elevated PC risk. However, *BRCA2* showed a PC Cluster Region (PCCR), specifically c.756–c1000 and c.7914+ with PSVs linking to elevated risk of disease [18].

A dearth of consensus pertaining to screening high-risk PC patients was prevalent [8]. To mitigate this, the IMPACT Study (Identification of Men with a genetic predisposition to PC: Targeted screening in *BRCA 1/2* mutation carriers and controls) screened and enrolled 1522 PC patients with germline *BRCA 1/2* mutation along with 959 controls [19] with annual prostate specific antigen (PSA) testing and warranting prostate biopsy if PSA >3ng/mL were performed. *BRCA2* carriers (3.3%) showed a higher incidence of PC than their *BRCA1* counterparts (2.6%) and controls (<2%) [16]. More than 67% of *BRCA2* and 61% of *BRCA1* carriers were classified under the intermediate/high-risk category.

### 1.2. Germline and Somatic Testing in Prostate Cancer

Integrative genomic profiling of prostate tumors has provided insights that improve the understanding and treatment of mCRPC. In 2015, the Cancer Genome Atlas (TCGA) evaluated 333 primary PC and concluded that alterations in DDR genes were common, as affected almost 20% of samples through mutations or deletions in BRCA1/2, CDK12, ATM, FANCD2, or *RAD51C* [20]. In terms of actionable genomic alterations in mCRPC, the AR pathway is the most frequently mutated (70%), followed by PI3K-AKT-mTOR pathway (40–60%), DDR (25%), and cell cycle regulators (25%) [4]. Given the availability of new synthetic lethal drugs, namely PARP inhibitors, the DDR pathway gene alterations have become particularly important to detect; among DDR genes, *BRCA2* is the most frequently mutated gene in PC [4]. Moreover, it is estimated that 8% of mCRPC harbor germline mutations with implications for family members of PC patients and genetic testing [4]. Frequency of germline mutations of DDR genes associated with PC has been investigated in 692 metastatic PC patients by Pritchard et al. [21] and in 419 mCRPC patients by Castro et al. (PROREPAIR-B) [22]. Both studies identified *BRCA2* as the most frequently mutated DDR gene in PC and identified DDR germline mutation at a frequency between 12 and 7.3% [21,22]. Interestingly, no family history or younger age at diagnosis were correlated to harboring a germline mutation of a DDR gene [15]. *BRCA2* mutation was found to associate with the worst outcome and shorter survival, with important clinical implications. Indeed, those with *BRCA2* mutations reached a cancer-specific survival of 17.4 months, as compared to the wild-type patients with the prolonged 33.2 months (*p* = 0.027) [22].

Based on the above-mentioned data, current recommendations from major oncological international societies (National Comprehensive Cancer Network (NCCN), American Society for Radiation Oncology (ASTRO), and European Association of Urology (EAU), ESMO) strongly invite clinicians to consider genomic testing for their PC patients. More recently, the Philadelphia PC consensus conference also recommended more specifically germline testing in all PC patients at any stage with broad gene panel or, if not available, at least gene testing in *BRCA1/2*, MMR genes [23].

However, several issues still need to be clarified, such as: (a) at which stage of the disease should the patients be tested (diagnosis, relapse, mCRPC), (b) the recommended tissue for the analysis, (c) if it is best to perform somatic or germline testing only or both [24,25,26,27,28]. Moreover, it should be answered whether circulating tumor DNA (ctDNA) can replace tumor tissue at any time point. With this regard, early studies have confirmed a remarkable concordance of ctDNA and metastatic tissue biopsies in mCRPC, suggesting that ctDNA assays could be confidently used to molecularly stratify patients for prognostic and predictive purposes [29,30]. Overall, most of the research ongoing in this field is mostly trying to shed light on these very important clinical concerns. For example, it has been shown that alteration frequency of typical PC mutations (i.e., *AR*, *PTEN*, *RB1*, *ATM*, *CDK12*, among others) progressively increases from locoregional disease to metastatic-non-castrate to castrate-resistant PC. This has implications from the clinical standpoint, if, for example, treatment decisions for a patient already treated with several lines of therapy are taken based on the results of gene sequencing performed on a diagnostic biopsy [31,32,33]. It seems that somatic *BRCA* mutations are more often observed in late stages of PC. As such, it is strongly recommended for a genomic re-assessment with a new solid or liquid biopsy for an updated snapshot of the tumor [34,35]. It has not yet been clarified whether to perform germline testing first, followed by somatic testing or vice versa; performing germline testing in all patients with PC would be cheaper and easier to implement but would miss approximately 50% of patients eligible for PARP inhibitors, whereas while implementing a somatic mutation, only testing would be more expensive and would risk missing identification of germline mutations.

Overall, germline data drive more aggressive screening in men at high risk of developing PC, whilst somatic testing is performed to determine whether the tumor has actionable targets for therapy. Prior knowledge of germline mutations can help in the interpretation of the results. Although tumor-based testing potentially identifies both germline and somatic mutations, it is unable to differentiate them. Somatic testing with target genes can be used as an initial screening test to provide personalized precision medicine to patients. This decreases the amount of time and resources spent on blood-based germline testing followed by tumor testing to identify a somatic mutation in the absence of germline mutations. Molecular tumor boards are needed to best interpret results and to direct clinical management and trial opportunities for providers and patients.

Another important issue that has emerged by past screening effort within pivotal trials (PROFOUND, TRITON2, and IPATENTIAL) is the high failure rate of next-generation sequencing (NGS) testing; between 30 to 50% of patients screened in these studies failed NGS testing. This has an implication for standard care testing of patients to be directed to target therapy in the future [33,36,37].

Sequencing of somatic mutations in tumor biopsies (primary prostate tissue or metastatic lesion) can use multigene panels and molecular profiling. There are several NGS panels used to compare mutations for different quantitative assessments of prognosis and resistance to therapy [38]. Moreover, several clinical germline multigene panels, specifically designed for PC patients, are currently used in the USA; all panels include *BRCA1* and *BRCA2* genes. The aim of such panels is to identify driver mutations and molecular targets, and to allow a personalized treatment of cancer. The understanding that men with mCRPC can harbor a mutation in the DNA repair pathway has been the basis for the development of trials that evaluate the clinical response of various clinical therapies. A report from the European Society for Medical Oncology (ESMO) Precision Medicine Working Group, which was recently published, used the scale of actionability to define the relative importance of mutations based on the availability of treatment options and evidence supporting their use [39]. The following genes were listed with the relative actionability level (ESCAT): *BRCA1/2* (1A), *MSI-H* (1C), *PTEN* (IIA), *ATM* (IIA), *PALB2* (IIB), *PI3KCA* (IIIA), *AKT* (IIIA). Tier I actionability indicates an alteration-drug match associated with improved outcome in clinical trials. Tier II is an antitumor activity associated with the matched alteration-drug but lacks prospective outcome data, while for Tier III, the matched drug-alteration leads to clinical benefit in another tumor type other than the tumor of interest. It is thus evident that, at present, *BRCA1/2* and *MSI-H* gene mutations represent the alterations with strongest therapeutic actionability and predictivity of therapeutic success [40].

### 1.3. Implications for the Treatment

Platinum-based chemotherapy alkylates DNA cause interstrand crosslinks; it is known that this type of DNA damage would lead to cell death in *BRCA* associated HR- deficient tumor cell [5,15]. Based on this rationale, satraplatin, a novel platinum agent, was used in a randomized phase III trial with mCRPC patients with prior progression to taxanes. Although risk of disease progression was reduced, it failed to show a benefit in overall survival (OS) over placebo [2,7]. The results of this trial led to limited usage of platinum salt in PC patients.

In the above context, platinum salts may not be a standard of care in PC, but their use is recommended in neuroendocrine differentiation [5]. 141 mCRPC patients were treated with carboplatin AUC 3–5 and docetaxel 60–75 mg/mq at the Dana Farber Cancer Institute between 2001 and 2015 [41]. 6 out of 8 of *BRCA2* carriers showed >50% decline in PSA levels at 12 weeks when compared with 23/133 or 17% of non-carriers. Such a decline was associated with longer OS, i.e., 18.9 months (carriers) vs. 9.5 months (non-carriers) [41]. Another study of 109 mCRPC patients evaluated efficacy of platinum-based chemotherapy after progression to taxanes; it showed high PSA decline (>50%) in patients with DDR alterations (50%) compared to DDR proficient ones (13%) confirming previous evidence of higher response and clinical benefit in patients with DDR gene defects [42]. Moving forward from satraplatin, the above results have undoubtedly generated interest in the resurgence of platinum-based chemotherapy in PC.

PARP proteins consist of enzymes which sense and repair SSBs and are involved in several other cellular processes including cell death. Their inhibition leads to formation of DNA replication forks, creating DSBs which would require repair by HR or NHEJ [43]. In a background of *BRCA* mutation, characterized by HR deficiency, NHEJ operates. The process, being prone to errors, prevails in cell cycle arrest and apoptosis [44]. This lethality caused by two separate genomic events, i.e., PARP inhibition and DDR-deficient state, occurring synchronously, is called synthetic lethality [45]. *BRCA* defective cells are therefore sensitive to PARP inhibitors [5,45].

The United States (US) Food and Drug Administration (FDA) has approved, for the treatment of mCRPC, the PARP inhibitors olaparib, rucaparib, and niraparib. They differ in terms of their metabolism; olaparib and rucaparib are metabolized by cytochrome P450 enzymes, whilst niraparib by carboxylesterase-catalyzed amide hydrolysis. The most common side effects include gastro-intestinal manifestations, myelosuppression, and fatigue. Importantly, the synthetic lethality may act against severe PARP inhibitors-mediated toxicity. PARP–DNA complexes have the ability to interfere with DNA replication, and PARP trapping has a role related to the cytotoxicity of PARP inhibitors. Based on that, the magnitude of cytotoxicity differs between PARP inhibitors. Olaparib, rucaparib, and niraparib trap PARP approximately 100-fold more efficiently as compared to veliparib [46]. Olaparib was the first PARP inhibitor showing significant activity in mCRPC cases with prior progression to standard treatment. Kaufman et al. evaluated 298 advanced cancer patients with germline *BRCA1/2* mutation in a single-arm phase II study; 8 of them were mCRPC, of which 7 harbored BRCA2 mutation and the remaining one, being a BRCA 1 mutant; the observed objective response rate (ORR) was 50% and disease control rate was 75% [47].

UK-based TOPARP (Trial of PARP inhibition in PC) phase II trial followed this observation, and it was conducted in two stages. The first stage (TOPARP-A) tested anti-tumor activity of olaparib in a sporadic mCRPC population [48]; the second stage (TOPARP-B) was conducted in patients with known genomic background, specifically *BRCA2* or *ATM* mutations to assess sensitivity to olaparib [49]. The primary endpoints were a composite endpoint including radiological response by RECIST (Response Evaluation Criteria in Solid Tumors) or PSA decline >50% or circulating tumor cell (CTC) count reduction from >5 to ≤5 CTC/7.5ml blood in at least 2 readings, four weeks apart. The secondary endpoints were progression-free survival (PFS) and OS. In TOPARP-A, 50 heavily pretreated mCRPC patients with docetaxel and abiraterone ± enzalutamide, received olaparib 400 mg twice a day [48]. 49 of them were assessed for response to olaparib; 16 out of the 49 patients had a response to olaparib (ORR 33%) on the basis of the composite definition of response specified in the study protocol. The antitumor activity was more pronounced when *BRCA1/2*, *ATM*, Fanconi’s anemia genes, and *CHEK2* mutations were present [49]. 14 out of the 16 (88%) achieved clinical benefit both in terms of radiological response and decline in PSA ± CTC. In TOPARP-B, among 92 mCRPC patients, half were administered olaparib, 300 mg twice daily, and the other half 400 mg twice daily [50]. Radiographic and PSA responses in the 400 mg cohort (54% and 24%) were superior to that of the then 300 mg cohort (39 and 16% cohort). 30 out of 98 cases were *BRCA1/2*-deficient and achieved 52% radiographic and 77% PSA response as compared to 5 and 11.3%, respectively, in other DDR defects (namely *ATM*, *CDK12*).

The phase III biomarker-driven PROfound Trial confirmed the association between DDR defects and PARP inhibitor response in PC, which led to approval of olaparib in this setting [33]. 387 patients with mCRPC, previously treated with AR signaling inhibitors were recruited into 2 cohorts; cohort A (included *BRCA 1/2*, *ATM* mutations) with 245 patients and cohort B (*BARD1*, *CDK12*, *CHEK1/2*, *FANCL*, *PALB2*, *RAD51A/B/C/D*, *RAD54L*, and other defects) with 142 patients. These patients were given olaparib 300 mg twice daily and second line AR signaling inhibitors in a 2:1 ratio. Radiological PFS (rPFS) was the primary endpoint. A median rPFS of 7.4 vs. 3.5 months and median OS of 19.1 vs. 14.7 months were observed in cohort A in patients treated with olaparib vs. AR signaling inhibitors, respectively. PROfound also showed a better efficacy of olaparib in *BRCA* mutants, especially *BRCA2* mutant, unlike other DDR defect groups. As previously mentioned, these results led the FDA to approve olaparib in mCRPC patients with germline or somatic HR repair mutations after progression on AR signaling inhibitor. Today, it is an approved modality in the US and Europe but not in the UK [2,5].

Two phase II trials, TRITON2 and GALAHAD, evaluating the efficacy of another two PARP inhibitors, namely rucaparib and niraparib, in heavily pretreated mCRPC patients who have shown progression on an AR signaling inhibitor and taxanes, have also been reported [36,51]. The primary endpoint was the ORR. The TRITON-2 trial enrolled 190 mCRPC candidates of which 98 had *BRCA1/2* defects whereas the rest had other germline or somatic DDR [26]. Rupacarib 600 mg twice daily was used. Radiological and PSA response, i.e., ORR, was higher in *BRCA* mutant patients (43.9%) than in *ATM* (9.5%) or other DDR mutant patients (0%). The GALAHAD trial enrolled 165 mCRPC patients with defined biallelic alterations in BRCA1/2, ATM, FANCA, PALB2, CHEK2, BRIP1 or HDAC2, who were treated with niraparib 300 mg twice daily. ORR (41 vs. 9%) and rPFS (8.2 vs. 5.6%) was higher in *BRCA*-deficient carriers than other DDR deficiencies [42/51]. PSA decline of greater than 50% was observed in 50% of patients with *BRCA1/2* and 3% of those with non-*BRCA* biallelic DDR gene alterations. Similar to olaparib, rucaparib was approved by the FDA for use amongst mCRPC patients with germline and/or somatic *BRCA1/2* mutations undergoing prior progression on AR signaling inhibitor or taxane. Europe still awaits approval [2,5]. Table 2 summarizes the characteristics of the PROfound, TRITON2, and GALAHAD studies in the mCRPC.

The combination of PARP inhibition and AR signaling inhibitors could represent another example of synthetic lethality. AR is a ligand-inducible transcription factor. AR signaling inhibitors or ADT block the AR signaling and lead to HR deficit or downregulated state. This in turn creates an upregulation of PARP which acts as a backup repair system [52]. Clarke et al. reported the results of a phase III randomized trial, which assessed the efficacy of combination therapy olaparib and abiraterone vs. placebo and abiraterone in mCRPC patients with prior docetaxel progression [53]. Although no significant differences were noted in ORR, time to radiographic progression was longer in olaparib arm (13.8 months) than control arm (8.2 months).

### 1.4. Immunotherapy in Prostate Cancer

Immune checkpoint therapies have recently revolutionized the treatment approach of several solid tumors including melanoma, and non-small cell lung cancers. Efficacy of these agents in PC has been disappointing so far. The two most validated immune checkpoint targets are cytotoxic T-lymphocyte-associated protein 4 (CTLA-4) and programmed cell death protein 1 (PD1) and its ligands (programmed death-ligand 1/2, PD-L1/L2). CTLA-4 is currently targeted by ipilimumab while PD1/PD-L1 by pembrolizumab, nivolumab, atezolizumab, and durvalumab. Beer et al. did a randomized, double-blind phase III trial where patients were randomly assigned to two groups: ipilimumab 10 mg/kg vs. placebo every 3 weeks for up to 4 doses. 399 patients were treated with ipilimumab, and 199 patients were treated with placebo. Median PFS and OS in ipilimumab arm were 5.6 and 28.7 months whereas in the placebo arm they were reported to be 3.8 and 29.7 months, respectively. OS, being the primary endpoint, was therefore not impacted but progression of disease was delayed [54]. The IMbassador250 phase III trial randomized 759 patients with mCRPC who underwent prior progression on abiraterone and docetaxel, or in whom ADT was not administered to atezolizumab (atezo) and enzalutamide (enza) (*n* = 379) vs. enza alone (*n* = 380). Primary endpoint was improvement in OS; median OS for atezo + enza vs. enza alone were 15.2 vs. 16.6 months, respectively, thus not meeting the primary endpoint [55].

Resistance to approved checkpoint inhibitors is currently believed to be related to the evidence that mCRPC tumors are inevitably immunologically “cold” probably due to their lower somatic mutation tumor burden with consequently reduced tumor-infiltrating T-cells. Combination therapy using multiple checkpoint inhibitors have been proposed to mount a potent T-cell response in PC and to potentially overcome intrinsic resistance to single agent checkpoint inhibition. The Checkmate 650 trial combined CTLA-4 (ipilimumab 3 mg/kg) and PD-L1 (nivolumab 1 mg/kg) in 90 patients with 45 each in cohort 1 (pre-chemotherapy) and cohort 2 (post-chemotherapy). The ORR, median PFS, median OS were 25%, 5.5 months, 19 months and 10%, 3.8 months, 15.2 months in cohort 1 and 2 respectively [56]. Results were promising as compared to the monotherapy counterparts. In line with other tumor types, MMR-deficient mCRPC patients have shown response to immune-checkpoint inhibitors, due to the accumulation of somatic mutations, and consequently, the high neoantigen burden. Pembrolizumab was approved by the FDA in 2017 for solid metastatic MMR-deficient tumors and can be used in MMR-deficient PC patients [57]. The KEYNOTE-199 study recruited 258 patients with prior progression on docetaxel and targeted endocrine therapy to receive pembrolizumab. Median OS was 9.5, 7.9, and 14.1 months in three cohorts of patients with PD-L1 positive, negative, and bone-predominant regardless of PD-L1 expression disease, respectively [58]. Ongoing and future biomarker studies from KEYNOTE-199, including gene expression profiles and tumor mutational burden, will define molecular markers of response to pembrolizumab. Loss-of-function alterations of tumor suppressor protein CDK12 was found in approximately 5–7% of PC. Translational studies demonstrated that *CDK12* mutations may delineate an immuno-responsive subgroup of PC with increased levels of T-cell infiltration and neoantigens. Based on that, *CDK12*-mutated tumors might constitute a separate subgroup of PC in which immunotherapy may be effective [59,60,61]. So far, the largest cohort of *CDK12*-inactivated PC patients treated with immunotherapy has been provided by two independent retrospective multicenter series. They have described the outcomes of 112 *CDK12*-mutated tumors in total [62,63]. Among them, 28 received diverse immunotherapy regimens and favorable responses were achieved even by some heavily pretreated cases. Several key conclusions can be made at that stage. These patients often present with high-risk features, including Gleason grade group 4–5, T3–T4 disease, and de novo metastases. Regardless of the biochemical response, the PFS on AR-signaling inhibitors was generally short. Moreover, responses to immune checkpoint blockade seem to be enriched in less heavily pretreated patients. Finally, recent correlate analysis of mCRPC biopsies revealed *CDK12*-mutated mCRPCs were enriched in immunosuppressive CD4+FOXP3- cells [64].

There are no FDA approved indications for immune checkpoint inhibitors for treatment of castrate-sensitive PC; however, their use is being evaluated in clinical trials. A phase III trial is underway to evaluate pembrolizumab plus enzalutamide plus ADT versus enzalutamide and ADT alone [NCT04191096]. Multiple phase I and phase II trials are evaluating immune checkpoint inhibitors in combination with treatments such as abiraterone and cabozantinib [NCT04477512], radiation therapy [NCT04262154, NCT03795207], and an experimental IL-8 directed monoclonal antibody [NCT03689699]. In addition, perioperative ipilimumab in combination with castration prior to radical prostatectomy has demonstrated feasibility with longer follow-up ongoing [65].

## 2. Conclusions and Future Directions

DNA sequencing efforts have changed the molecular classification of prostate tumors and are leading to precision medicine strategies as well as defined prognosis and clinical features of molecular subsets of PC. Nevertheless, prospective studies demonstrating clinical value of biomarkers for prognostication or prediction of response are warranted. Somatic and germline DNA testing for patients with advanced PC should be considered in view of the therapeutic consequences for the patient and the possibility of pursuing targeted screening in this population. Metastatic tumor biopsies are recommended to obtain information regarding mCRPC tumor features. Germline samples are easily collected and analyzed, but still half of the patients with somatic DDR defects would not be identified. Liquid biopsies may be used to monitor for the detection of secondary mutations that may restore the function of a gene previously altered.

PARP inhibitors represent one of the recent biggest therapeutic developments for PC patients. Combination of PARP inhibitor with AR-targeting agents is worthy due to the cross-regulation of both pathways and the central role of hormonal therapy in PC. Immunotherapy has shown still limited efficacy in these patients. However, immunotherapy combinations will probably overcome intrinsic resistance of PC to immunotherapy. Ongoing studies that assess the role of immunotherapy in PC are NCT04104893, NCT04019964 and NCT03570619.

## Figures and Tables

**Table 1:** Single-nucleotide polymorphisms and germline DDR gene mutations in PC.

Pathway	Genes	Clinical Impact
BER	*XRCC1*	Increased risk
NER	*XPC, XPD, XPG,* and *CSB*	
MMR	*MSH5*	
HR	*BRCA1/2*	Increased risk, aggressive biological behavior, early onset, nodal involvement
	*RAD51B* and *BRIP1*	Increased risk
	*NBS1*	Aggressive biological behavior
NHEJ	*XRCC4*	Increased risk
	*XRCC6*	Increased risk, aggressive biological behavior
	*MVP*	

gDDR: germline DNA damage repair; PC: prostate cancer; HR: homologous recombination; BER: base excision repair; NER: nucleotide excision repair; MMR: mismatch repair; NHEJ: non-homologous end joining.

**Table 2:** Principal PARP inhibitors’ monotherapy studies in mCRPC.

	PROfound	TRITON2	GALAHAD
Phase	III	II	II
Agent	Olaparib	Rucaparib	Niraparib
Dosage	300 mg b.i.d.	600 mg b.i.d.	300 mg q.d.
Previous Treatment	ARS inhibitors	ARS inhibitors and taxane	
Specimen Tested	Tumor tissue	Plasma or tumor tissue	Plasma
Primary Objective	rPFS in patients with alterations in *ATM* and *BRCA1/2*	ORR and PSA response in patients with DDR alterations	ORR in patients with biallelic *BRCA1/2*

PARP: poly (ADP-ribose) polymerase; mCRPC: metastatic castration resistant prostate cancer; b.i.d.: bis in die; q.d.: quaque die; ARS: androgen receptor signaling inhibitors; rPFS: radiological progression-free survival; ORR: objective response rate; DDR: DNA damage repair.
